# Harnessing the potential role of lignocellulolytic microbial consortia for rapid decomposition of rice straw

**DOI:** 10.3389/fmicb.2026.1825309

**Published:** 2026-04-24

**Authors:** A. Anveshitha, Sonth Bandeppa, Sodimalla Triveni, Kanuri Komala Siva Katyayani, N. Hari Sathwika, K. Shailaja, V. Manasa, Nallagatla Vinod Kumar, P. C. Latha, Venkateswarlu Ronda, Debasis Mitra, R. M. Sundaram, M. B. B. Prasad Babu

**Affiliations:** 1 Professor Jayashankar Telangana Agricultural University, Hyderabad, Telangana, India; 2 Indian Institute of Rice Research, Hyderabad, Telangana, India; 3 Indian Institute of Millets Research, Hyderabad, Telangana, India; 4 Department of Microbiology, Graphic Era (Deemed to be University), Dehradun, Uttarakhand, India

**Keywords:** carbon sequestration, lignocellulolytic, microbial consortia, rice straw, soil fertility, sustainable intensification, waste valorization

## Abstract

**Introduction:**

Agricultural residue burning represents a critical nexus between food security and climate crisis, with rice straw combustion alone contributing 141.15 Mt CO_2_ equivalent emissions annually in India. This study addresses this issue by developing a microbial consortium approach to convert agricultural waste into a carbon-sequestering soil amendment, simultaneously mitigating climate impact and soil degradation.

**Methods:**

Microbial cultures were isolated from cow dung, partially degraded straw, and forest soil, and screened for lignocellulolytic enzyme activity. Three efficient isolates were identified via 16S rRNA and ITS gene sequencing as *Bacillus subtilis* (IIRRSDB-6), *Pseudomonas aeruginosa* (IIRRSDB-7), and *Mariannaea camptospora* (IIRRSDF-3). These isolates were selected to develop a microbial consortium and evaluated under in vitro conditions for lignocellulose degradation efficiency.

**Results:**

The microbial consortium exhibited strong enzymatic synergy, with *B. subtilis* showing peak lignin peroxidase activity (3.177 U/mL) and laccase production (4.245 U/mL), while *M. camptospora* recorded maximum laccase activity (5.547 U/mL). Rice straw decomposition was significantly enhanced, reducing the carbon-to-nitrogen ratio from 66.24 to 19.45 within 60 days (70.6% improvement). Nutrient enrichment was observed, with nitrogen increasing from 0.7 to 1.62% (131%), phosphorus from 0.17 to 0.28% (64.7%), and potassium from 1.53 to 2.09% (36.6%). Structural biomass degradation included reductions in acid detergent fiber (40.3%), cellulose (41.3%), and lignin (55.7%).

**Conclusion:**

The developed microbial consortium effectively transforms agricultural residues into nutrient-rich, carbon-sequestering soil amendments. This provides a scalable and sustainable alternative to residue burning, promoting waste valorization, soil fertility improvement, and climate change mitigation.

## 1 Introduction

Rice (*Oryza sativa* L.), a crop deeply intertwined with the sustenance of billions and the cultural fabric of Asia where over 90% is cultivated, presents a paradox. Its monumental global production of 523.9 million tons in 2023 (Statista, 2024), cultivated across an astonishing 120,000 cultivars worldwide, secures global food security. Yet, this success generates a significant challenge: the disposal of rice straw, a voluminous agricultural residue that often meets a destructive end. In the critical agricultural heartlands of the northwestern Indo-Gangetic plains of India, encompassing states like Punjab, Haryana, and Uttar Pradesh, the sheer scale of rice straw production, coupled with the deeply entrenched practice of open-field burning, has ignited a multi-faceted environmental and public health crisis. The seasonal ritual of burning an estimated 23 million tons of rice stubble, compressed within a narrow window dictated by subsequent wheat planting (NAAS, 2017), unleashes a catastrophic plume of greenhouse gases, black carbon, and hazardous particulate matter (Roy, 2022), severely degrading air quality, exacerbating respiratory ailments, and contributing significantly to climate change. We recognize the logistical constraint posed by the 30-day window available between rice harvest and subsequent wheat planting in the Indo-Gangetic Plains (IGP). While our current *in vitro* findings span 60 days, data collected at the 30-day mark demonstrate a rapid reduction in both carbon content (30.38% reduction) and recalcitrant lignin (ADL 6.26% from 9.01%). This accelerated rate indicates the consortium's high enzymatic activity potential, suggesting that with optimized field application, substrate preparation, and solid-state fermentation strategies, the system can be adapted to meet the critical 30 days decomposition requirement. This destructive practice not only inflicts immediate environmental harm but also undermines the long-term sustainability of agriculture by depleting essential soil nutrients, disrupting soil microbial communities, and reducing overall soil health (Gupta et al., 2004; Jat et al., 2013; Sahu et al., 2015). The imperative for innovative and ecologically sound rice straw management strategies is therefore undeniable. While microbial decomposition offers a promising pathway toward sustainable residue management, current approaches frequently fall short of achieving the desired efficiency and efficacy. Existing composting techniques are often too slow and labor-intensive for practical implementation on a large scale, while the application of single microbial strains or poorly defined microbial mixtures often lacks the enzymatic breadth and synergistic interactions necessary for the comprehensive degradation of the recalcitrant lignocellulosic components of rice straw, limiting nutrient release and hindering the creation of valuable soil amendments.

This study boldly departs from conventional wisdom by introducing a fundamentally novel approach: implementation of a functionally optimized microbial consortium tailored specifically for climate-positive rice straw management. Instead of relying on serendipitous single microbe, we have strategically developed a consortium comprised of *Bacillus subtilis* (IIRRSDB-6), *Pseudomonas aeruginosa* (IIRRSDB-7), and *Mariannaea camptospora* (IIRRSDF-3), each meticulously selected for its unique and complementary roles in the degradation of cellulose, hemicellulose, and lignin the primary structural components of rice straw. The defining characteristic of this consortium is its rational design, guided by a rigorous and multi-faceted selection process based not only on quantitative enzymatic activity assays but also on genomic analysis to predict potential synergistic interactions and metabolic complementarity. To further enhance the novelty, the consortium members were not sourced from conventional agricultural soils but rather from unique and under-explored environmental niches cow dung, partially degraded straw, and forest soil thus maximizing the probability of identifying novel and highly specialized lignocellulolytic microbes required for synergistic consortium action. We hypothesize that the precisely orchestrated synergistic interactions between the rationally selected strains will unlock unprecedented levels of decomposition efficiency, specifically achieving a C:N ratio below 20:1 and a lignin reduction exceeding 50% within 60 days under controlled conditions, thereby accelerating nutrient mobilization, promoting the formation of stable soil organic matter, and ultimately shifting the rice straw cycle from a source of environmental pollution to a driver of soil health and climate resilience. Moving beyond mere quantification of decomposition rates, this study presents a comprehensive and integrated analysis of the consortium's impact on a wide range of key chemical, physical, and biological soil parameters, including detailed measurements of carbon sequestration, greenhouse gas emissions, and changes in soil microbial community structure. This research transcends the conventional narrative of microbial decomposition by providing a tangible pathway toward climate-positive agriculture, demonstrating how a rationally designed microbial consortium can efficiently transform rice straw into a valuable soil amendment, mitigate the negative impacts of residue burning, enhance soil health, sequester carbon, and contribute to a more sustainable and resilient agricultural future. This study offers a blueprint for future research in this area, demonstrating the power of a holistic approach that considers not only decomposition efficiency but also the broader environmental and economic benefits of microbial-mediated rice straw management.

## 2 Materials and methods

Sixty isolates (Anveshitha et al., 2023) were evaluated for lignocellulolytic activity via cellulase and ligninase enzyme assays by employing both qualitative and quantitative plate assays.

### 2.1 Qualitative assay

#### 2.1.1 Screening for cellulolytic activity

Cellulase activity was evaluated using carboxy methyl cellulose (CMC) agar and Reese's mineral media assays. On the CMC agar assay (Teather and Wood, 1982), wells were created in agar (pH 7.2 ± 0.2) containing carboxymethyl cellulose, inoculated with pure cultures, and incubated at room temperature for 3–5 days. Plates were stained with 0.1% Congo red and de-stained with 1 M NaCl; clear halos around the wells indicated cellulase activity. The Reese's mineral media assay (Mandels and Reese, 1957) was performed using Reese's mineral media containing 1.1% CMC, incubated at 30 °C for bacterial and 27 °C for fungal cultures. After 5 days, the plates were treated with Congo red staining followed by destaining with NaCl. The formation of yellow zones around colonies, confirming cellulase production.

#### 2.1.2 Screening for ligninolytic activity

Lignin peroxidase activity was assessed on minimal media supplemented with 0.01% Azure B, where decolorization zones around colonies after incubation at 30 °C indicated enzyme production. Similarly, minimal media containing 0.01% methylene blue (Sasikumar et al., 2014) were used to screen bacterial and fungal isolates incubated at 27 °C for 2 weeks. The decolorization of surrounding colonies confirmed lignin peroxidase activity. Laccase activity of fungal isolates was evaluated using tannic acid, 2-2′-azino-bis(3-ethylbenzothiazoline-6-sulfonic acid) (ABTS), and guaiacol-based plate assays. In the tannic acid plate assay, isolates were grown on minimum media (pH 5.5) supplemented with tannic acid (Ander and Eriksson, 1977) and incubated at 30 °C for 3 days. The development of dark yellow to brown zones around colonies indicated tannin degradation and confirmed laccase activity. 2-2′-Azino-bis(3-ethylbenzothiazoline-6-sulfonic acid) (ABTS) oxidation assay was performed using media containing 0.1% 2-2′-azino-bis(3-ethylbenzothiazoline-6-sulfonic acid) (Pointing, 1999), with incubation at 30 °C for 3 days, the formation of a violet halo around the colonies confirmed, indicative of laccase production. For the guaiacol plate assay, isolates were cultured on low salt medium with 0.01% guaiacol (Sharma et al., 2017) at 27 °C for 2 weeks. Reddish-brown coloration around colonies further validated laccase activity and ligninolytic potential.

### 2.2 Quantitative assay

The activity of lignin peroxidase (LiP) was assessed utilizing the H_2_O_2_ dependent oxidation of Azure B technique (Andlar et al., 2018). The assay combination comprised 0.5 mL of enzyme filtrate, 0.5 mL of citrate buffer (pH 4.8), 50 μL of Azure B (0.05 M), and 50 μL of H_2_O_2_ (10 mM). Absorbance at 651 nm was measured every 30 s for a duration of 180 s. Enzyme activity was quantified as the quantity inducing a 0.01-unit variation in absorbance per minute per mL. A control devoid of H_2_O_2_ was used as a reference. Laccase activity was measured using 5 mM 2-2′-azino-bis(3-ethylbenzothiazoline-6-sulfonic acid) (ABTS) as the substrate, in accordance with the method outlined by Yin et al. (2019). The reaction mixture comprised 1 mL of enzyme filtrate, 1 mL of citrate buffer (0.05 M, pH 4.8), and 0.2 mL of 5 mM ABTS (made by dissolving 0.027 g ABTS in 10 mL distilled water). The assay commenced with the addition of ABTS and whereas the reference cuvette excluded ABTS. Absorbance at 436 nm was recorded at 30 s intervals for a duration of 180 s. Enzyme activity was quantified as the quantity that induces a 0.01 unit alteration in absorbance per minute per mL.

### 2.3 Molecular identification of potential lignocellulolytic isolates

The genomic DNA of the selected microbial isolates was extracted using the GSURE^®^ bacterial genomic DNA isolation kit (GCC Biotech). Amplification of 16S rRNA genes (for bacterial isolates) and ITS genes (for fungal isolates) was conducted using a Bio-Rad T100 Thermal Cycler (Bio-Rad, USA). The primers used for 16S rRNA were: 8F: 5′-AGAGTTTGATCCTGGCTCAG-3′; 907R: 5′-CCGTCAATTCCTTTRAGTTT-3′; 785F: 5′-GGATTAGATACCCTGGTA-3′; 1492R: 5′-CGGTTACCTTGTTACGACTT-3″ (Lane, 1991). For the amplification of fungal ITS, the following primers were used: ITS-1F: 5′-CTT GGT CAT TTA GAG GAA GTA-3′; ITS-4: 5′-TCC TCC GCT TAT TGA TAT GC-3″. The amplified products (16S rRNA for bacteria: approximately 1,500 bp; ITS for fungi: approximately 600 bp) were sequenced using the Sanger sequencing. The sequences were analyzed using the CAP3 BioEdit (CAP3 was developed by Xiaoqiu Huang and Anup Madan at Michigan Technological University, Houghton, MI, USA; BioEdit was developed by Thomas A. Hall at North Carolina State University, Raleigh, NC, USA) software to construct contigs. The consensus sequences obtained were compared with the National Center for Biotechnology Information (NCBI) GenBank nucleotide database using BLAST to identify the isolates based on sequence similarity.

### 2.4 Compatibility study between bacterial and fungal isolates with each other

The compatibility of bacterial isolates was assessed using the cross-streak technique on a nutrient agar medium. Bacterial isolates were streaked at right angles and cultured for 4 days at 30 °C. After incubation, the isolates were assessed for the presence of inhibitory zones. Isolates that displayed no inhibition zones were considered compatible. Compatibility of fungal and bacterial isolates was evaluated using the dual culture plate method, as outlined by Velho-Pereira and Kamat (2011).

### 2.5 Development of the microbial consortium

An effective microbial consortium was developed by isolating and identifying promising rice straw-degrading microorganisms based on their lignocellulolytic activity assessed through cellulase and ligninase enzyme assays, followed by optimization of their compatibility and evaluating their degradation efficiency.

### 2.6 Evaluation of selected promising lignocellulolytic isolates for rice straw degradation

The study assessed the decomposition of rice straw (Improved Samba Mahsuri variety) during a 60 days period under *in vitro* conditions at the ICAR-Indian Institute of Rice Research (IIRR), Hyderabad. The experiment was conducted by inoculating the three individual strains and consortia, along with the control and four replications. The moisture level was maintained at 60% during composting with the addition of water.

Treatments:

T_1_-Control (straw without microbe).

T_2_-*Bacillus subtilis* + straw.

T_3_-*Pseudomonas aeruginosa* + straw.

T_4_-*Mariannaea camptospora* + straw.

T_5_-Microbial consortia + straw.

#### 2.6.1 Analysis of straw chemical composition

The carbon content of rice straw was estimated using the dry combustion method by gravimetric measurement of the loss of carbon. A finely powdered sample of 1–5 g of rice straw was placed in crucible, and the crucible with sample was kept in a muffle furnace for 1 h at 550 °C, after which it was removed cooled, weighed and the final weight was calculated the ash percentage. The carbon content was calculated from ash % (Baruah and Barthakur, 1997).

Samples were examined at 0, 15, 30, 45, and 60 days for N, P and K concentrations, carbon-to-nitrogen (C:N) ratio, cellulose, and lignin. Nitrogen was quantified using the Kjeldahl method, phosphorus was quantified using the Vanado-molybdo yellow color method, and potassium was quantified using a flame photometer. The C:N ratio was determined using dry combustion and ash analysis. The acid detergent fiber (ADF), acid detergent lignin (ADL) and cellulose contents were determined by methods described by Van Soest et al. (1991) using Fibretherm instrument. ADF represents the cellulose and lignin content, whereas ADL represents the lignin content of rice straw. The decomposition rate of the rice straw was determined by measuring the reduction in straw weight over time.

### 2.7 Statistics

The experiments were conducted using a completely randomized design (CRD), and all samples were processed using four biological replicates. The findings were analyzed using one-way analysis of variance (ANOVA) to assess significance or differences using, SPSS version 16 and R software. Statistical significance was set at *p* < 0.05, according to the Duncan's multiple range test.

## 3 Results and discussion

### 3.1 Qualitative assay

#### 3.1.1 Screening for cellulolytic activity

**Figure 1:**
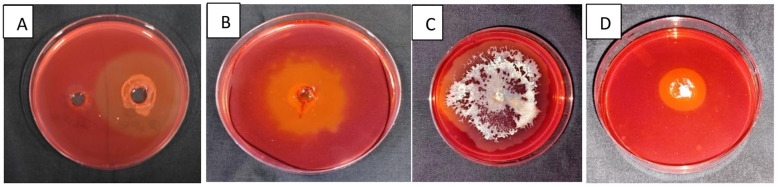
Screening for cellulolytic activity: **(A, B)** Zone of hydrolysis on CMC agar plates, **(C, D)** Zone of hydrolysis of fungal isolates on Reese's media.

Sixty isolates were examined on carboxymethyl cellulose (CMC) agar plates, which demonstrated distinct zones of hydrolysis in 21 bacterial and two fungal isolates, signifying cellulase activity. The hydrolysis capacity (HC) values varied from 2.58 (CDB-2) to 1.00 (FSB-5), with 23 isolates exhibiting HC > 1.0, indicating significant cellulolytic activity (Figure 1). These results are consistent with those of Saini et al. (2017), who highlighted the application of halo zones as indicators of cellulase activity. Mahmood et al. (2020) showed cellulolytic activity in 24 of 42 bacterial isolates. Enhanced cellulase synthesis in bacteria is due to their rapid growth and metabolic adaptability (Chen et al., 2024). Polysaccharides such as cellulose, have the potential to bond with the dye to form a visible complex on the plate; however, when cellulose breaks down into mono and disaccharides around the colony, they are unable to bind efficiently with the dye, resulting in a clear zone. Among the 15 fungal isolates evaluated on Reese's mineral medium, three (CDF-3, CDF-4, and PDSF-3) showed distinct zones surrounding their colonies, indicating cellulase production (Figure 1). This aligns with the findings of Bairagi (2016), who detected cellulase activity in 52 fungal isolates. Fungi are recognized for their capacity to generate extracellular enzymes, that are essential for cellulose degradation (Viji and Neelanarayanan, 2015).

#### 3.1.2 Screening for ligninolytic activity

Fifteen isolates (11 bacterial and four fungal) demonstrated lignin peroxidase activity through the decolorization of Azure B dye. The microbial isolates displayed varying decolorization zones, with a few isolates showed higher activity toward Azure B dye. The decolorization of Azure B was explicitly associated with strong redox potential agents like lignin peroxidases (Dube et al., 2023). Kaur et al. (2016) reported analogous findings, noting lignin peroxidase activity in bacterial and fungal strains under comparable circumstances. The results were augmented by Riyadi et al. (2022), who screened three isolates *Aeribacillus* sp., *Bacillus subtilis* and *Stenotrophomonas* sp., for ligninase enzyme activity on minimal media with Azure B as the substrate. Azure B was determined to be a more specific indicator of lignin peroxidase activity (LiP) as its decolorization requires strong oxidative enzymes like LiP; conversely, methylene blue served as a broader indicator, as it can also be decolorized by other oxidative enzymes such as manganese peroxidase (MnP) and laccase.

Five isolates demonstrated ligninolytic capacity by decolorizing methylene blue dye. These findings correlate with those of previous studies that reported, lignin peroxidase activity in bacterial isolates derived from cow dung and soil (Bandounas et al., 2011; Dube et al., 2023). Sasikumar et al. (2014) reported that eight bacterial isolates *Pseudomonas* sp. expressed decolorization of methylene blue which showed ligninase activity. Accordingly, methylene blue is a thiazine that can be broken by high redox potential oxidase enzymes, such as lignin peroxide (Archibald, 1992).

##### 3.1.2.2 Laccase activity

**Figure 2:**
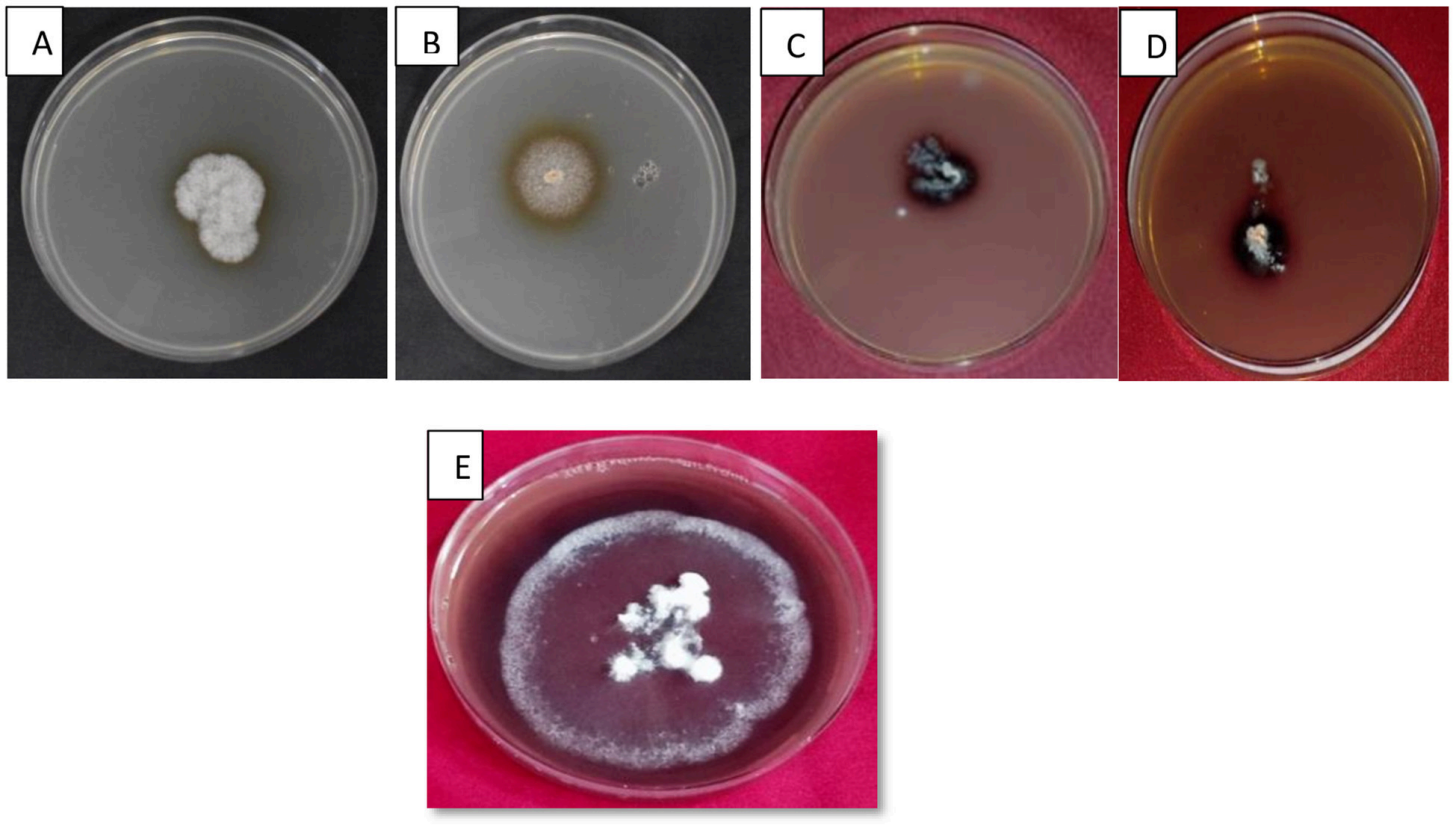
Screening for ligninolytic activity: **(A, B)** Laccase activity brown colorization on minimal media supplemented with tannic acid. **(C, D)** Laccase activity reddish brown colorization on guaiacol plate. **(E)** Laccase activity violet colorization on 2-2′-azino-bis(3-ethylbenzothiazoline-6-sulfonic acid (ABTS) plate.

The 7 days old culture of four fungal isolates (CDF-3, CDF-4, CDF-5, and PDSF-3) demonstrated laccase activity within 72 h of incubation at 30 °C. Isolates formed dark yellow to brown zones in media supplemented with tannic acid (Figure 2). Laccase oxidizes the phenolic compound tannic acid which leads to the formation of a brown zone around the colony. Sharma et al. (2017) identified tannic acid oxidation as a dependable indicator of laccase synthesis. Similarly, Silva et al. (2010) reported that filamentous fungi, particularly *Aspergillus niger* and *Penicillium* sp., which have ligninolytic activity were grown with tannic acid as the sole carbon source. Comparable results were reported by Shinde et al. (2022) *Trichoderma* and *Aspergillus* showed brown zones on media plates indicating laccase enzyme production.

ABTS oxidation was observed only in one isolate CDF-3, resulting in violet zones surrounding the colonies (Figure 2). This activity is indicative of laccase production, as demonstrated by More et al. (2011) and Shakhova and Volobuev (2020), who employed ABTS as a substrate to assess the oxidative enzyme activity. Laccase activity has been widely observed in fungi such as *Trametes versicolor, Pleurotus ostreatus*, and *Ganoderma lucidum* (D'Souza-Ticlo et al., 2009), highlighting its role in lignin degradation and bioremediation. The positive oxidation of CDF-3 indicates its potential for industrial and environmental applications.

**Figure 3:**
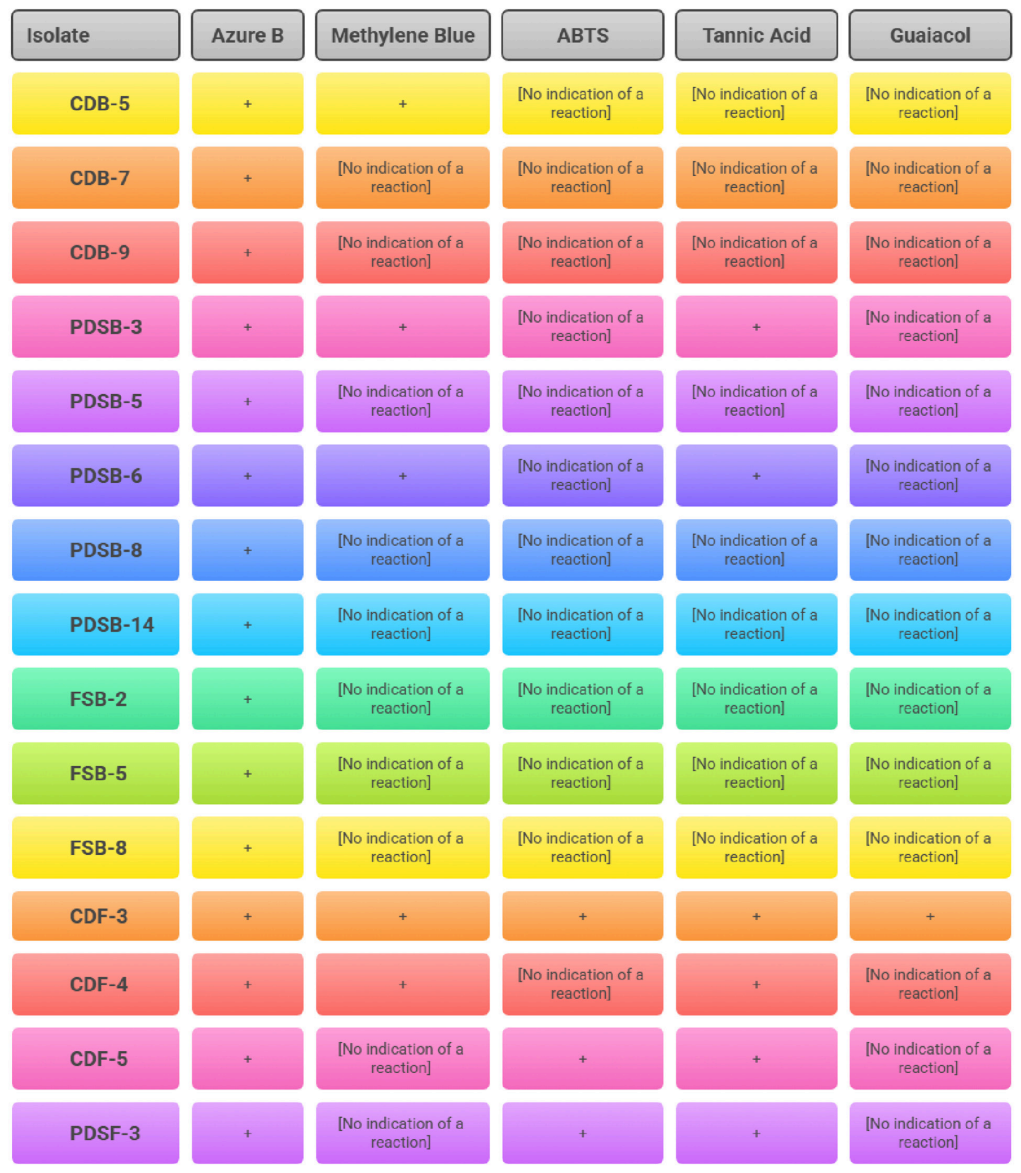
Qualitative assay of ligninolytic enzyme activity.

Five isolates (CDF-3, CDF-5, PDSF-3, PDSB-3, and PDSB-6) revealed reddish-brown zones, thereby demonstrating laccase activity (Figure 2). The qualitative assay of ligninolytic enzyme activity represented (Figure 3). Guaiacol oxidation serves as an indicator of ligninolytic potential, as evidenced by Fu et al. (2013), who reported analogous activity in lignocellulolytic fungi. The results obtained were in line with previous studies reported by Atalla et al. (2010), where seven isolates showed brown zones indicating laccase enzyme activity. Sayyed et al. (2020) described *Aspergillus* sp. producing brown halos around mycelium on guaiacol agar plates due to ligninolytic enzyme activity, including laccase, confirmed by oxidation of guaiacol and ABTS substrates. This showed glucose as a carbon source increased laccase production significantly, supporting its role in enzyme activity detection via color changes around colonies. Abd El Monssef et al. (2016) used PDA supplemented with 0.04% guaiacol to screen of laccase producing fungi and observed a potent laccase producer *Trichoderma harzianum*.

### 3.2 Quantitative assay

#### 3.2.1 Lignin peroxidase activity

**Figure 4:**
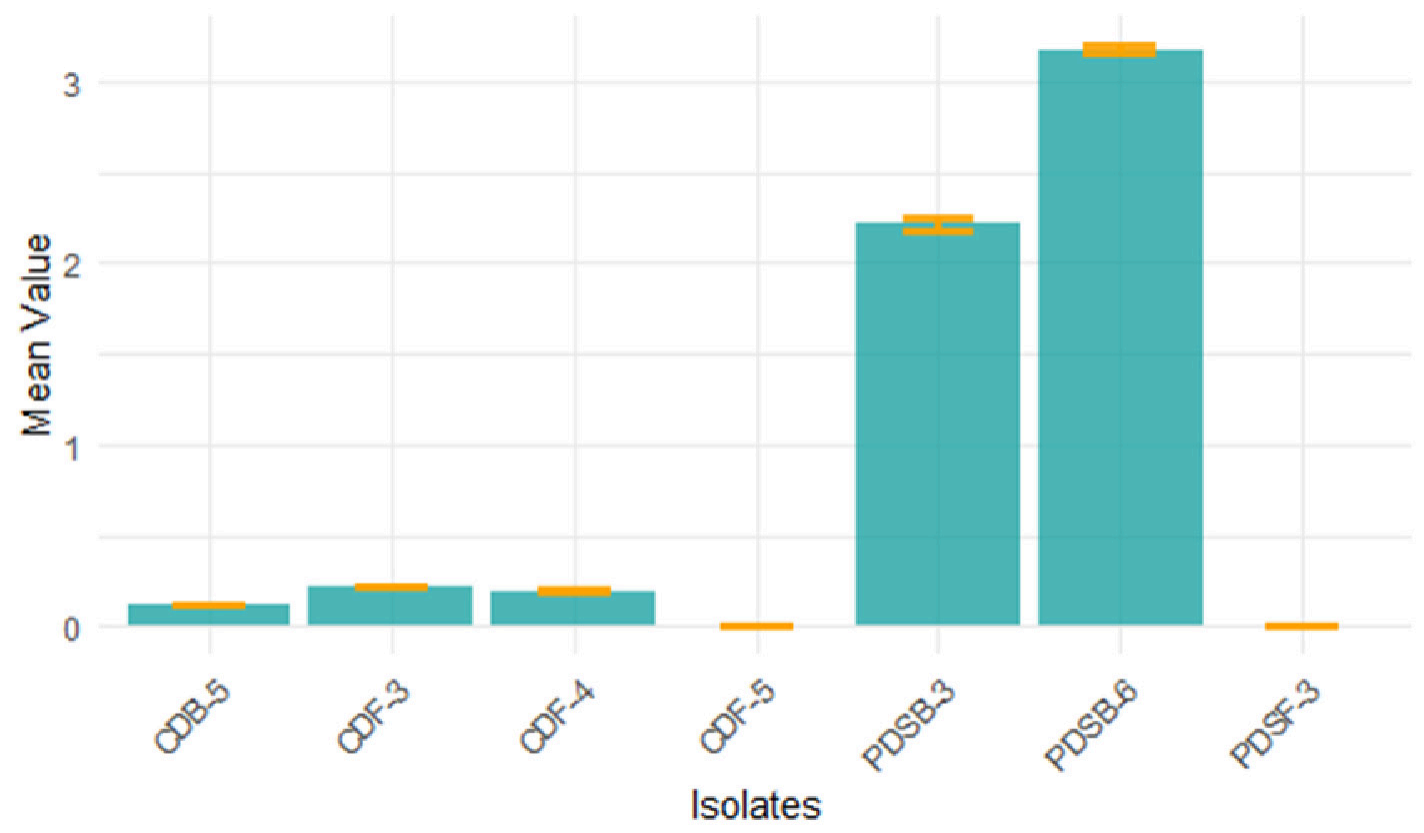
Quantitative analysis of lignin peroxidase activity between 0.119 U/mL and 3.177 U/mL throughout the five microbiological isolates.

Quantitative analysis of lignin peroxidase indicated an enzyme activity between 0.119 and 3.177 U/mL throughout the five microbiological isolates studied. The highest lignin peroxidase activity was recorded for PDSB-6 at 3.177 U/mL (Figure 4), with a specific activity of 1973.29 U/mg protein. This was followed by PDSB-3 at 2.214 U/mL and 1064.42 U/mg, CDF-3 at 0.218 U/mL and 120.44 U/mg. Meanwhile CDF-4 and CDB-5 produced lowest lignin peroxidase activity which was recorded at 0.195, 0.119 U/mL, with a specific activity of 79.59 U/mg, 70.83 U/mg. The results were represented (Table 1). Lignin peroxidase, a glycosylated extracellular enzyme that demands H_2_O_2_, is essential for the degradation of complex lignin polymers into simple molecules. The elevated enzyme activity found in PDSB-6 corresponds with the findings of Rahman et al. (2013), who showed the maximum production of lignin peroxidase in *Bacillus subtilis*. Banakar and Thippeswamy (2014) documented similar lignin peroxidase activities in *Pleurotus* sp. and *Phanerochaete chrysosporium*. The results indicate that these microbial isolates have potential for lignin degradation in the decomposition of agricultural residues, making them a prospective option for biotechnological applications in agro-waste management.

**Table 1:** Quantitative assay of ligninolytic enzyme.

Isolate name	lignin peroxidase assay		laccase assay	
	Enzyme activity (U/mL)	Specific activity (U/mg)	Enzyme activity (U/mL)	Specific activity (U/mg)
CDB-5	0.119	70.83	-	-
PDSB-3	2.214	1064.42	5.074	1307.73
PDSB-6	3.177	1973.29	4.245	2345.30
CDF-3	0.218	120.44	5.547	2579.70
CDF-4	0.195	79.59	-	-
CDF-5	-	-	3.498	1169.90
PDSF-3	-	-	2.278	886.38

#### 3.2.2 Laccase activity

**Figure 5:**
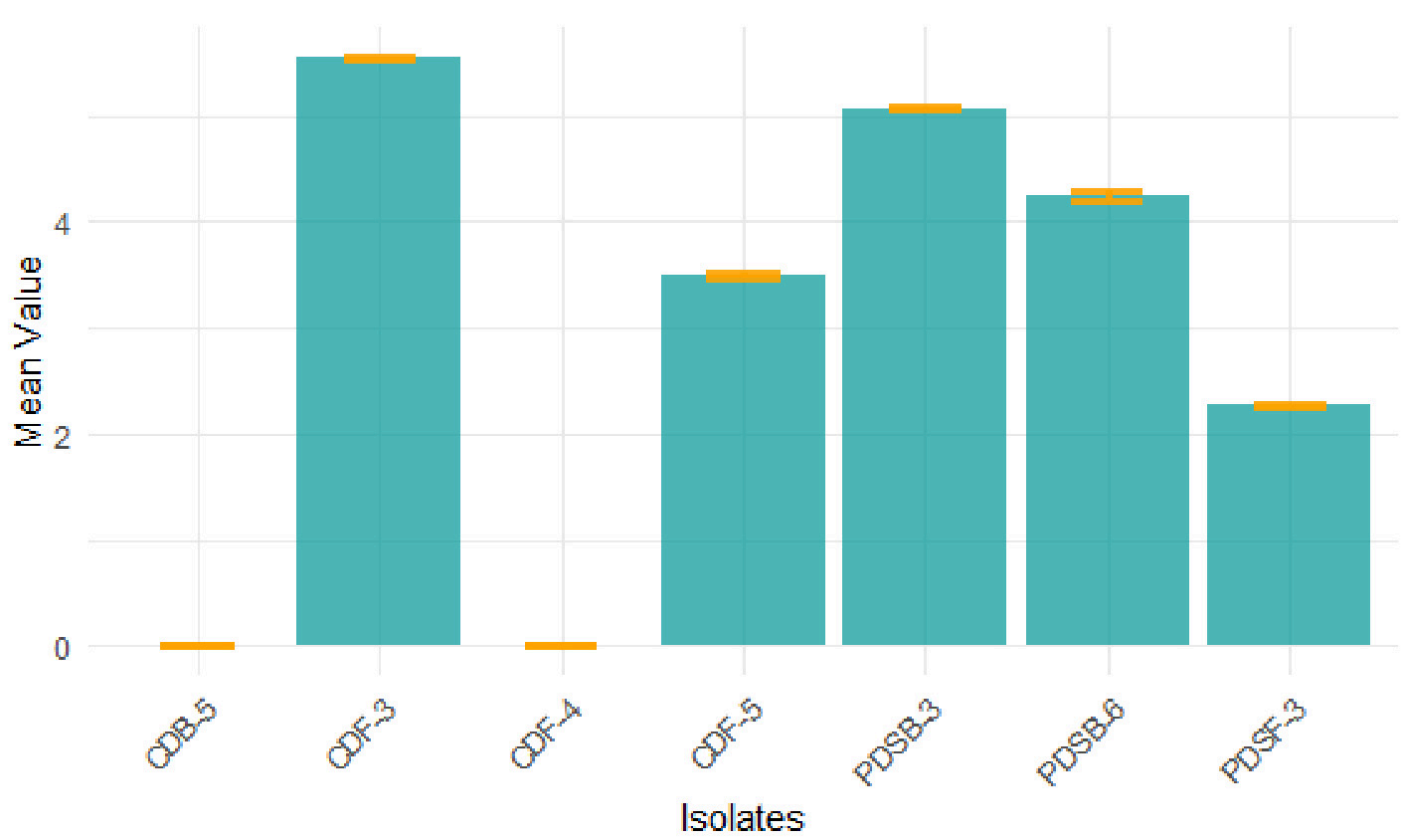
Quantitative study of Laccase activity with ABTS as a substrate indicated activity levels between 2.278 U/mL and 5.547 U/mL.

A quantitative study of laccase production with ABTS as a substrate indicated activity levels between 2.278 and 5.547 U/mL (Figure 5). The greatest amount of enzyme activity was seen in CDF-3 (5.547 U/mL), exhibiting a specific activity of 2579.70 U/mg protein, followed by PDSB-3 (5.074, 1307.73 U/mg), PDSB-6 (4.245, 2345.30 U/mg), CDF-5 (3.498, 1169.90 U/mg), and PDSF-3 (2.278, 886.38 U/mg). The results are presented in Table 1. Laccase, a multi-copper oxidase, is crucial for lignin breakdown, facilitating the oxidation of both phenolic and non-phenolic lignin subunits. These results align with Sharma et al. (2017), who documented laccase activity ranging from 1.12 to 3.42 U/mL in *Trichoderma* sp. and *Aspergillus* sp. The enhanced efficacy of CDF-3 highlights its potential for lignin degradation and biofuel generation, especially in the treatment of rice straw residues.

The integrated assessment of lignin peroxidase and laccase activities underscored the ligninolytic efficacy of the selected isolates. PDSB-3, PDSB-6, and CDF-3 were distinguished as superior performers for efficient lignin breakdown. These results confirm the findings of Sarma et al. (2022), who highlighted the significance of ligninolytic enzymes in expediting the decomposition of agricultural residues. The detected enzymatic activities indicate that these isolates may be essential for creating microbial consortia to improve rice straw decomposition and foster sustainable agricultural practices.

#### 3.2.3 Mechanistic basis for superior enzymatic performance

The differential enzymatic performance observed is attributed to the specialized metabolic profiles of the isolates. *Bacillus subtilis* (PDSB-6), which recorded the maximum lignin peroxidase activity (3.177 U/mL), is widely recognized for its high capacity to produce ligninolytic enzymes. Crucially, *Mariannaea camptospora* (CDF-3), the top laccase producer (5.547 U/mL), represents a significant finding due to this species being severely underexplored in agricultural waste management. The high efficacy of CDF-3 in lignin degradation, supported by its exceptional acid detergent fiber (ADF) reduction capacity (35.90%), suggests the possession of highly specialized enzymatic machinery for targeting the recalcitrant cellulose-lignin complex. The strategic combination of these complementary strengths high LiP from the bacterium and peak laccase from the fungus forms the rational basis for the consortium's overall enhanced decomposition efficiency.

Future applications must prioritize the optimization of enzyme synthesis via environmental and substrate-specific changes. Furthermore, amalgamation of these isolates into a microbial consortium may provide synergistic effects, as evidenced by Wan and Li (2012), who highlighted enhanced lignocellulosic degradation by multi-enzyme-producing microbial consortia. This method can resolve agricultural waste management issues and facilitate biofuel manufacturing.

### 3.3 Molecular identification and evolutionary relationships

Molecular characterization was conducted for 12 potential lignocellulolytic microbial isolates, including eight bacterial (CDB-5, CDB-9, PDSB-3, FSB-2, PDSB-2, PDSB-6, CDB-7, and PDSB-8) and four fungal (CDF-3, CDF-4, CDF-6, and PDSF-3) isolates. The PCR products were visualized by using agarose gel electrophoresis, and the sequencing results were matched against the NCBI GenBank database for identification. The isolates were deposited in GenBank, and accession numbers were obtained. The bacterial isolates were identified as *Bacillus, Serratia, Pseudomonas*, and *Cytobacillus*, where the fungal isolates included *Trichoderma harzianum, Metarhizium carneum, Mariannaea camptospora*, and *Aspergillus flavus*. The bacterial isolates included three *Bacillus* species: IIRRSDB-1 (*Bacillus* sp., 96% similarity, GenBank OR553095), IIRRSDB-2 (*Bacillus cereus*, 99.54% similarity, OR553102), and IIRRSDB-6 (*Bacillus subtilis*, 100% similarity, OR546350). Additional bacterial isolates comprised IIRRSDB-3 (*Serratia marcescens*, 100% similarity, OR544799), IIRRSDB-4 (*Bacillus velezensis*, 99.66% similarity, OR563917), and IIRRSDB-7 (*Pseudomonas aeruginosa*, 100% similarity, OR544959). The application of 16S rRNA and ITS gene sequencing for microbial identification corresponds with research by Wang et al. (2023) and Sharma et al. (2017), which emphasizes its dependability in microbial taxonomy and ecological investigations.

**Figure 6:**
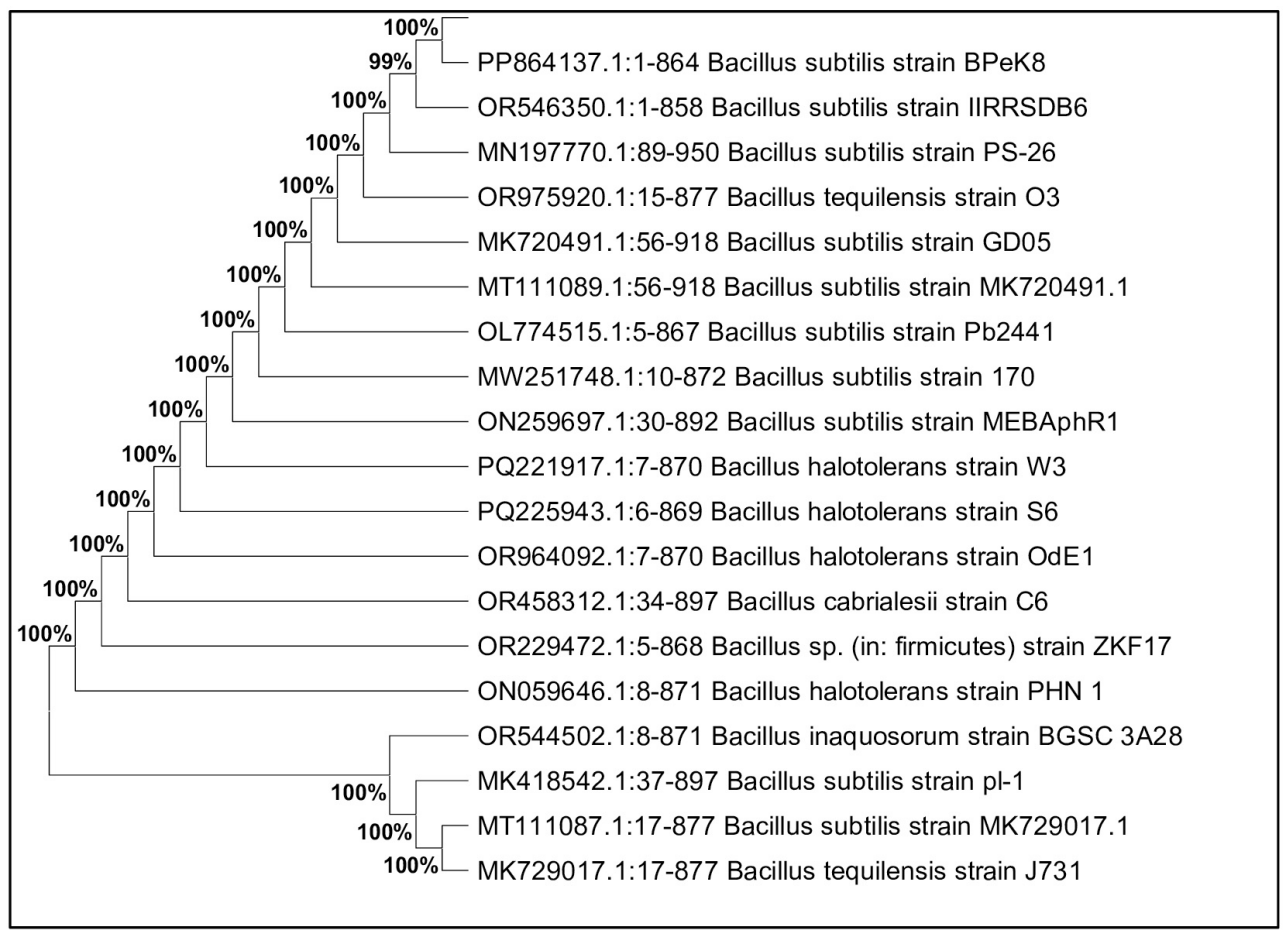
Phylogenetic position of strain *Bacillus subtilis* IIRRSDB-6.

**Figure 7:**
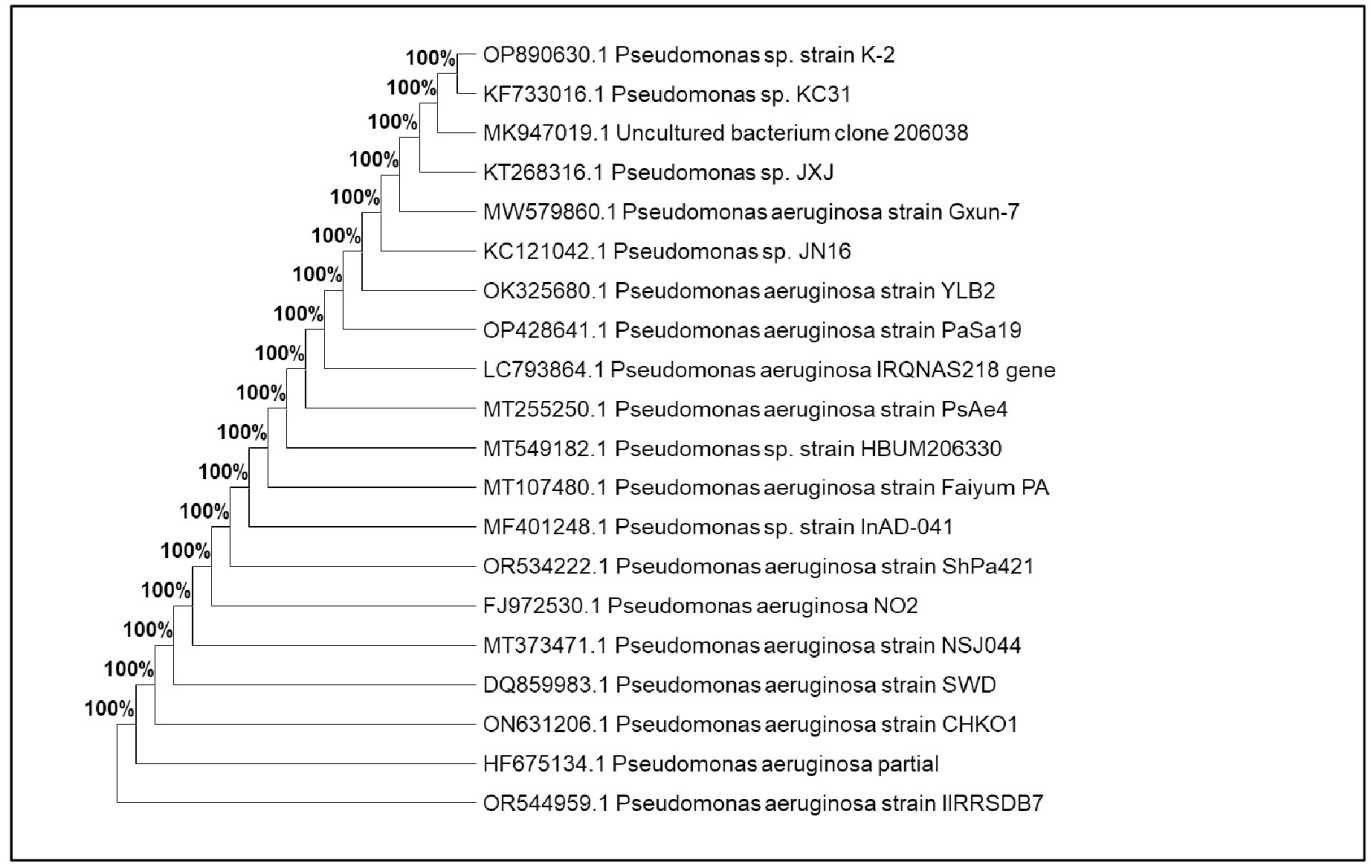
Phylogenetic position of strain *Pseudomonas aeruginosa* Isolate IIRRSDB7.

The constructed phylogenetic trees based on 16S rRNA sequences revealed interesting evolutionary clustering patterns that provide insights into functional enzyme evolution. Members of the *Bacillus* genus are widely recognized for their ability to produce cellulolytic and hemicellulolytic enzymes (Chen et al., 2024). Similarly, IIRRSDB-2 (*Bacillus cereus*) showed 99.54% similarity (OR553102), and this species is known for its ability to degrade lignocellulosic biomass efficiently (Kaur et al., 2016). IIRRSDB-3 (*Serratia marcescens*) with 100% similarity (OR544799) has been reported to produce a broad spectrum of hydrolytic enzymes, making it a promising candidate for agricultural waste decomposition. IIRRSDB-4 (*Bacillus velezensis*, OR563917) and IIRRSDB-6 (*Bacillus subtilis*, OR546350) with 99.66% and 100% similarity, respectively (Figure 6), are known for their biocontrol potential and lignocellulolytic capabilities (Sharma et al., 2017). Notably, *Pseudomonas aeruginosa* (IIRRSDB-7, OR544959) with 100% similarity (Figure 7) demonstrated lignin-degrading potential through lignin peroxidase (Harshita et al., 2018).

**Figure 8:**
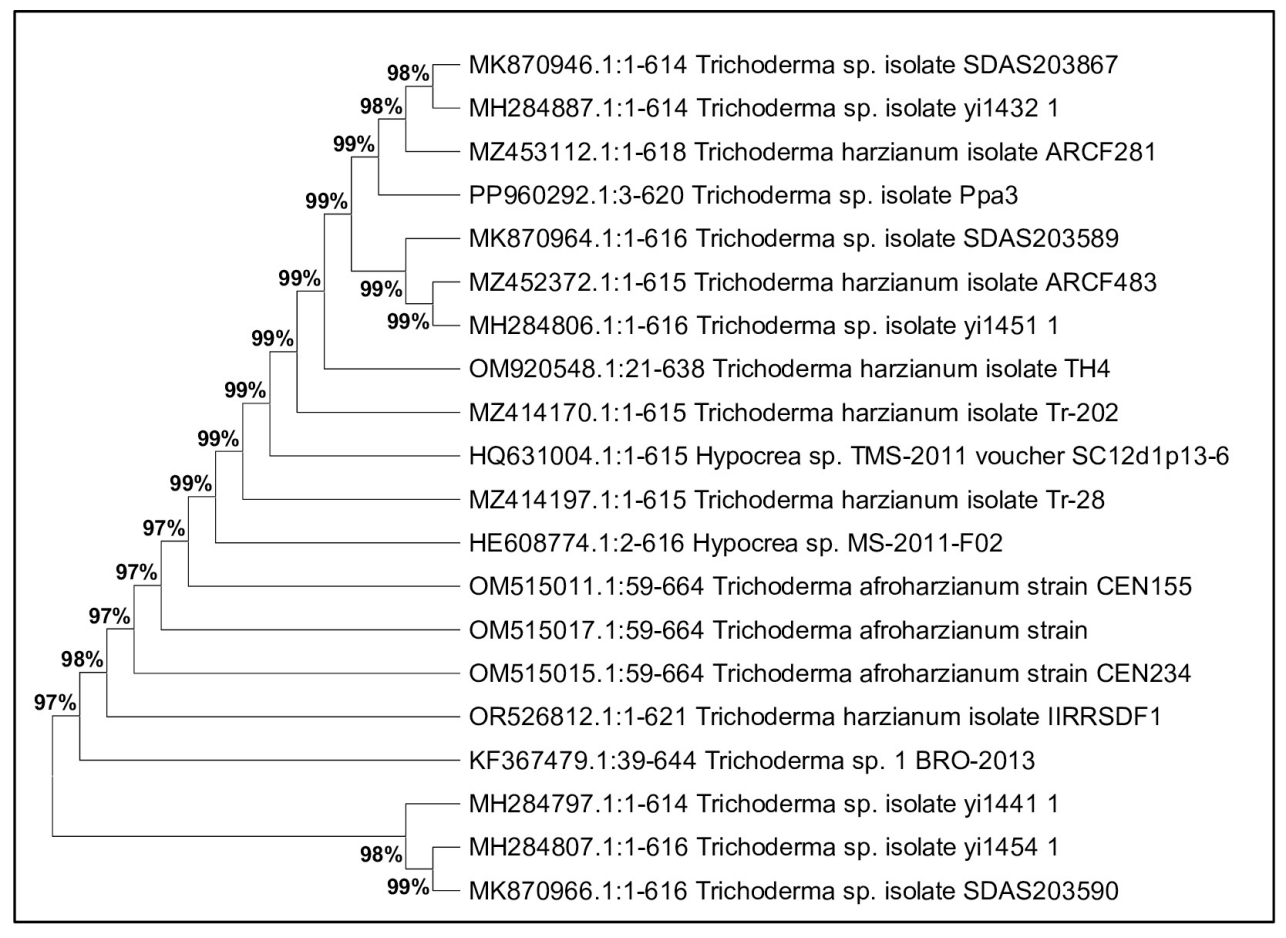
Phylogenetic position of strain *Trichoderma harzianum* Isolate IIRRSDF1.

Fungal identification revealed four taxonomically diverse species: IIRRSDF-1 (*Trichoderma harzianum*, 99.35% similarity, OR526812), IIRRSDF-2 (*Metarhizium carneum*, 99.40% similarity, OR533556), IIRRSDF-3 (*Mariannaea camptospora*, 99.18% similarity, OR530397), and IIRRSDF-4 (*Aspergillus flavus*, 99.25% similarity, OR530399). The fungal isolate IIRRSDF-1 (*Trichoderma harzianum*, OR526812) showed 99.35% similarity and is well-documented for its cellulase and ligninase production (Figure 8), critical for lignocellulosic degradation (Sarma et al., 2022). The strain IIRRSDF-2 (*Metarhizium carneum*, OR533556) demonstrated both insecticidal properties and production of lignocellulolytic enzymes, highlighting its dual functional potential. Additionally, *Mariannaea camptospora* (IIRRSDF-3, OR530397) and *Aspergillus flavus* (IIRRSDF-4, OR530399) were identified as robust producers of ligninase and cellulase, substantiating their utility in rice straw decomposition processes (Chen et al., 2024).

The identification of *Mariannaea camptospora* as a potent lignocellulolytic organism represents a significant finding, as this species has been severely underexplored in agricultural waste management applications despite its documented occurrence in lignocellulosic environments. Previous studies have primarily focused on its role in plant pathology, but our results demonstrate remarkable ADF reduction capacity (35.90%), suggesting specialized enzymatic machinery for cellulose-lignin complex degradation that warrants comprehensive biotechnological investigation (Huang et al., 2024).

### 3.4 Microbial consortium development and compatibility assessment

Compatibility studies among bacterial isolates using the cross-streak method, whereas interactions between bacterial and fungal isolates were evaluated using the dual culture method. The analyses aimed to assess the feasibility of creating efficient microbial consortia for the breakdown of rice straw, presenting a sustainable option compared with traditional disposal techniques. These findings indicated that all bacterial isolates expressed compatibility, as demonstrated by the lack of inhibitory zones, signifying the absence of antagonistic interactions. Similarly, the bacterial and fungal isolates demonstrated compatibility in the dual culture method, as no inhibitory zones were noted around the colonies. These results indicate that the microbial isolates are capable of coexisting in a harmonious manner, facilitating synergistic interactions that improve the efficiency of lignocellulose degradation. The compatibility of microbes plays a vital role in the performance of consortia, especially in processes that necessitate collaborative degradation of intricate substrates such as rice straw. Interactions among microbes, including mutualism and commensalism, foster enzymatic synergy, that boosts the production of cellulases, ligninases, and hemicellulases (Chen et al., 2024). When these enzymes work together cellulose, hemicellulose, and lignin are broken down into more accessible forms.

Harshita et al. (2018) reported comparable findings when, assessing the compatibility of *Pseudomonas fluorescens, Bacillus subtilis*, and *Trichoderma harzianum*. The study employed PDA plates and observed no antagonistic activity, thereby confirming that bacterial and fungal isolates can coexist without inhibiting each other. This compatibility promotes collaborative metabolic processes, leading to enhanced efficiency of lignocellulose breakdown. Sarma et al. (2022) underscored the potential of microbial consortia in accelerating up lignocellulose degradation, stressing the significance of compatibility in improving enzymatic efficiency. Moreover, microbial consortia offer synergistic interactions that outcompete single strains in decomposing complex substrates. Species such as *Aspergillus, Trichoderma*, and *Bacillus* are known to enhance decomposition by secreting a cocktail of enzymes and improving microbial succession (Sagarika et al., 2022). Engineered consortia and omics-guided formulations have shown promise in large-scale biomass valorization (Janeeshma et al., 2024). The creation of compatible microbial consortia is important for the sustainable management of rice straw. The formation of this consortium can improve substrate colonization and nutrient recycling, while also decreasing decomposition time, providing an environmentally friendly option for straw burning (Sharma et al., 2017). The transformation of agricultural residues into compost, biofertilizers, and bioenergy shows the role of microbial consortia in promoting resource conservation and fostering environmental sustainability.

### 3.5 Rice straw degradation kinetics and biogeochemical transformations

#### 3.5.1 Carbon dynamics and mineralization patterns

The *in vitro* degradation studies demonstrated remarkable efficiency of the microbial consortium in rice straw decomposition, revealing complex carbon transformation patterns previously unreported in agricultural waste management literature. Lignocellulolytic microbes significantly decreased the carbon content of the straw. At 30 days after inoculation (DAI), the highest reduction in the carbon was observed in (consortia + straw) resulted in 30.38%, when compared with (control + straw) 44.51% from the initial value 46.37% (Table 2). The decrease in the carbon content in (consortia + straw) from 30 DAI to 60 DAI was lesser 30.38% to 27.42%, this shows initially there was a rapid breakdown easily available carbon compounds in straw, later which showed slow rate due to complex carbon compounds. Similar findings were reported by Goyal and Sindhu (2011), who observed a 17.4% carbon reduction in rice straw treated with fungal consortia. The reduction in carbon content was due to microbial decomposition, leading to CO_2_ evolution and energy release (Adani et al., 1997), which is an indicating of enhanced organic matter decomposition and nutrient cycling (Nielsen et al., 2024). Straw carbon degradation rate (*k*-value) were also calculated in the present study. Treatments with microbial inoculants (consortia + straw) and (*Mariannaea camptospora* + straw) recorded elevated carbon degradation rates (*k*-value) compared to the control. This was related to the varied enzymatic activity of the microbial consortia, which efficiently degrade carbonaceous materials (Kumar et al., 2008; Sen et al., 2010).

**Table 2:** Effect of lignocellulolytic microbial inoculation on C contents and C:N ratio of straw under *in vitro* conditions.

Treatments	Carbon content (%)					C:N ratio				
	0 DAI	15 DAI	30 DAI	45 DAI	60 DAI	0 DAI	15 DAI	30 DAI	45 DAI	60 DAI
T_1_	46.37	45.335^a^ ± 0.4831	44.5125^a^ ± 0.3071	43.5^a^ ± 0.1476	43.4125^a^ ± 0.4313	66.24	61.0628^a^ ± 0.8357	54.4562^a^ ± 0.8355	44.2537^a^ ± 3.3168	41.3053^a^ ± 2.0022
T_2_	46.37	43.7475^b^ ± 0.1857	43.5825^b^ ± 0.4038	37.8375^c^ ± 0.2243	35.6325^c^ ± 0.392	66.24	44.7658^c^ ± 0.8836	39.3554^c^ ± 0.5982	30.1199^c^ ± 1.8713	25.682^c^ ± 0.3369
T_3_	46.37	44.75^a^ ± 0.4015	44.4075^a^ ± 0.2199	39.995^b^ ± 0.5741	38.285^b^ ± 0.984	66.24	51.7665^b^ ± 1.552	47.8822^b^ ± 0.5041	36.5067^b^ ± 2.7223	30.2776^b^ ± 1.1466
T_4_	46.37	42.2125^c^ ± 0.1438	32.47^c^ ± 0.2183	30.915^d^ ± 0.0569	28.5875^d^ ± 0.2686	66.24	39.1901^d^ ± 2.3109	27.1164^d^ ± 0.3131	22.4599^d^ ± 1.3043	19.134^d^ ± 0.6459
T_5_	46.37	39.865^d^ ± 0.9581	30.3775^d^ ± 0.1524	29.1075^e^ ± 0.3961	27.4175^e^ ± 0.347	66.24	33.6107^e^ ± 2.2719	21.9337^e^ ± 0.186	19.3083^d^ ± 0.7849	16.9772^e^ ± 0.2222

Values mentioned are the mean of four replications; DAI, days after inoculation; C:N ratio, carbon and nitrogen ratio.

Different small letters after mean values represent statistical differences (*P* = 0.05) among control (straw without microbe), Bacillus subtilis + straw Pseudomonas aeruginosa + straw, Mariannaea camptospora + straw, microbial consortium + straw; mean values followed by S.D. (±).

The C:N ratio significantly decreased with microbial inoculation. The C:N ratio of the straw was reduced from 0 DAI to 60 DAI in all treatments. However, the highest degree of reduction in the C:N ratio was observed in (consortia + straw) 66.24 to 16.98, followed by (*Mariannaea camptospora* + straw) 19.13, and (*Bacillus subtilis* + straw) 25.68, in contrast to T1 (control + straw) which had a C:N ratio of 41.31 (Table 2). The decrease in the C:N ratio of the straw might be due to the degradation of lignocellulosic residues (Kumar et al., 2008). Similar results were observed by Dash et al. (2022) showed a decrease in the C:N ratio from the first day of composting (60.0 to 67.3%) to the 28th DAC days after composting (DAC) (24.8 to 27.7%) treatment with *Bacillus cereus, Enterobacteriaceae bacterium, Penicillium* sp., *Alternaria alternata*. Fungal consortia enhanced decomposition by reducing carbon and increasing nitrogen availability (Bernal et al., 2009).

#### 3.5.2 Nutrient mineralization and soil fertility enhancement

The nitrogen (N), phosphorus (P), and potassium (K) contents of rice straw were assessed at 15, 30, 45, and 60 days after inoculation. Significant increases in N, P, and K were observed with microbial treatments, with the (consortia + straw) showing the highest increases. Nitrogen (N) increased in all the treatments, with (consortia + straw) reaching 1.62% after 60 days. Phosphorus (P) and potassium (K) contents also increased, with (consortia + straw) showing the highest values (0.28% and 1.83%, respectively; Tables 3, 4). These results align with those of previous studies, confirming that microbial treatments improve the nutritional value of crop residues, enhance soil fertility and reduce chemical fertilizer dependency (Muzamil et al., 2020). According to studies of Shukla et al. (2009) observed significantly increase in the NPK values during rice straw degradation inoculated with cellulolytic microbes, N content increased by 1.1%, P content increased by 0.22%, enhanced microbial activity facilitated nutrient mineralization. Lakshmi et al. (2014) highlighted that microbial consortium, including bacterial and fungal cultures, play a key role in phosphorus mobilization. Sharma et al. (2023) reported that fungal hyphae bind the available K with cellulose material because of their absorptive nature, which aids in raising K levels in composting. The observed increase in the NPK content in microbial-treated rice straw indicates that microbial degradation promotes the release of vital nutrients, thereby enhancing sustainable agricultural practices. The observed nutrient improvements result from multiple coordinated processes: (1) enzymatic breakdown of protein-bound nitrogen compounds by bacterial proteases, (2) phosphate solubilization through organic acid production, (3) potassium mobilization via structural component degradation, and (4) improved nutrient retention through microbial biomass formation. The demonstrated 131% increase in nitrogen content (from 0.7 to 1.62%) resulting in a final C:N ratio of 16.98 is particularly relevant. This high intrinsic N mineralization capacity suggests that this consortium may mitigate the necessity for initial nitrogen supplementation often required for decomposing high C:N residues, offering a significant cost saving and environmental advantage in field applications.

**Table 3:** Effect of single and consortia of lignocellulolytic microbial inoculation on NPK content of straw under *in vitro* conditions.

Treatments	Nitrogen content (%)					Phosphorous content (%)				
	0 DAI	15 DAI	30 DAI	45 DAI	60 DAI	0 DAI	15 DAI	30 DAI	45 DAI	60 DAI
T_1_	0.70	0.7425^e^ ± 0.0096	0.8175^e^ ± 0.0096	0.9875^c^ ± 0.0768	1.0525^e^ ± 0.0411	0.17	0.18^b^ ± 0.0163	0.1825^c^ ± 0.0126	0.1^e^ ± 0.0082	0.2^d^ ± 0.0082
T_2_	0.70	0.9775^c^ ± 0.0171	1.1075^c^ ± 0.0096	1.26^b^ ± 0.0816	1.3875^c^ ± 0.0096	0.17	0.2^b^ ± 0.0082	0.31^a^ ± 0.0668	0.22^c^ ± 0.0082	0.225^c^ ± 0.0173
T_3_	0.70	0.865^d^ ± 0.0252	0.9275^d^ ± 0.0096	1.1^c^ ± 0.0816	1.265^d^ ± 0.0238	0.17	0.19^b^ ± 0.0082	0.185^c^ ± 0.0058	0.2^d^ ± 0.0163	0.205^d^ ± 0.0129
T_4_	0.70	1.08^b^ ± 0.0653	1.1975^b^ ± 0.0096	1.38^b^ ± 0.0816	1.495^b^ ± 0.037	0.17	0.2^b^ ± 0.0163	0.225^bc^ ± 0.0129	0.24^b^ ± 0.0163	0.25^b^ ± 0.0082
T_5_	0.70	1.19^a^ ± 0.0816	1.385^a^ ± 0.0058	1.51^a^ ± 0.0816	1.615^a^ ± 0.0129	0.17	0.23^a^ ± 0.0163	0.255^b^ ± 0.0129	0.26^a^ ± 0.0082	0.275^a^ ± 0.0058

Values mentioned are the mean of four replications; DAI, days after inoculation. Different superscript letters (a–e) indicate statistically significant differences (*P* = 0.05) among Control (Straw without microbe), Bacillus subtilis + Straw, Pseudomonas aeruginosa + Straw, Mariannaea camptospora + Straw, Microbial consortia + Straw. Values are means ± standard deviation.

**Table 4:** Effect of single and consortia of lignocellulolytic microbial inoculation on K content of straw under *in vitro* conditions.

Treatments	Potassium content (%)				
	0 DAI	15 DAI	30 DAI	45 DAI	60 DAI
T_1_	1.53	1.57 ± 0.0816	1.63^e^ ± 0.0245	1.69^c^ ± 0.0816	1.795^c^ ± 0.0129
T_2_	1.53	1.7 ± 0.1633	1.75^c^ ± 0.0082	1.72^c^ ± 0.1633	1.71^d^ ± 0.0082
T_3_	1.53	1.62 ± 0.0816	1.69^d^ ± 0.0082	1.825^bc^ ± 0.0957	1.78^c^ ± 0.0082
T_4_	1.53	1.71 ± 0.0816	1.7925^b^ ± 0.0096	1.88^ab^ ± 0.0653	1.915^b^ ± 0.048
T_5_	1.53	1.83 ± 0.1633	1.9175^a^ ± 0.0096	1.99^a^ ± 0.0082	2.085^a^ ± 0.0191

Values mentioned are the mean of four replications; DAI, days after inoculation. Different superscript letters (a–e) indicate statistically significant differences (*P* = 0.05) among Control (Straw without microbe), Bacillus subtilis + Straw, Pseudomonas aeruginosa + Straw, Mariannaea camptospora + Straw, Microbial consortia + Straw. Values are means ± standard deviation.

#### 3.5.3 Lignocellulosic structure degradation analysis

##### 3.5.3.1 Acid detergent fiber (ADF) reduction

Lignocellulolytic microbial inoculation significantly reduced the ADF content of straw, where ADF representing cellulose and lignin, decreased significantly over 60 days. The lowest ADF observed in (consortia + straw) at 59.85% to 33.54%, followed by *Mariannaea camptospora* also reduced ADF by 35.90%, and (*Bacillus subtilis* + straw) at 38.11%. Higher ADF levels were recorded in (*Pseudomonas aeruginosa* + straw) at 40.14%, and the highest in the (control) at 56.11% (Table 5). Similar results were reported by Datsomor et al. (2022) who reported that straw treated with microbial consortium showed better degradation with an ADF value of (39.9%) when compared with control rice straw ADF value (55.7%). *Bacillus subtilis* and *Mariannaea camptospora* have shown effective lignin and cellulose breakdown, facilitating straw decomposition (Islam et al., 2021).

**Table 5:** Effect of single and consortia of lignocellulolytic microbial inoculation on ADF and lignin content of straw under *In vitro* conditions.

Treatments	ADF value (%)					Lignin content (%)				
	0 DAI	15 DAI	30 DAI	45 DAI	60 DAI	0 DAI	15 DAI	30 DAI	45 DAI	60 DAI
T_1_	59.85	59.1585^a^ ± 0.0312	58.2016^a^ ± 0.0453	57.1745^a^ ± 0.1424	56.1098^a^ ± 0.02	9.01	8.8529^a^ ± 0.0565	8.3983^a^ ± 0.1315	7.9963^a^ ± 0.0716	7.6548^a^ ± 0.0893
T_2_	59.85	57.1575^b^ ± 0.7881	50.838^c^ ± 0.5114	45.0734^c^ ± 0.342	38.1067^c^ ± 0.1748	9.01	8.4238^c^ ± 0.0694	7.9211^c^ ± 0.0648	7.4396^c^ ± 0.0894	6.8156^c^ ± 0.0607
T_3_	59.85	57.8775^b^ ± 0.7091	52.445^b^ ± 0.6136	46.0436^b^ ± 0.3566	40.1422^b^ ± 0.3992	9.01	8.6137^b^ ± 0.0673	8.1057^b^ ± 0.0013	7.7437^b^ ± 0.064	7.0549^b^ ± 0.0258
T_4_	59.85	56.0981^c^ ± 0.6922	49.5298^d^ ± 0.4107	43.5362^d^ ± 0.2993	35.9039^d^ ± 0.5318	9.01	7.8038^d^ ± 0.0573	7.0666^d^ ± 0.0201	6.1129^d^ ± 0.0524	5.0793^d^ ± 0.0556
T_5_	59.85	54.6968^d^ ± 0.3568	47.6447^e^ ± 0.5145	39.5035^e^ ± 0.3196	33.5378^e^ ± 0.395	9.01	7.479^e^ ± 0.1429	6.2628^e^ ± 0.0155	5.3299^e^ ± 0.1562	3.9899^e^ ± 0.0261

Values mentioned are the mean of four replications; DAI, days after inoculation; ADF, acid detergent fiber. Different superscript letters (a–e) indicate statistically significant differences (*P* = 0.05) among Control (Straw without microbe), Bacillus subtilis + Straw, Pseudomonas aeruginosa + Straw, Mariannaea camptospora + Straw, Microbial consortia + Straw. Values are means ± standard deviation.

##### 3.5.3.2 Lignin degradation

The ADL value of straw representing only lignin, was assessed at 0, 15, 30, 45, and 60 DAI with lignocellulolytic microbes significantly decreased the ADL value. The lowest significant decrease in value was in (control), initial ADL value of straw was 9.01 %, whereas at 15, 30, 45, and 60 DAI they recorded the values were 8.85%, 8.40%, 8.00%, 7.65%, followed by (*Pseudomonas aeruginosa* + straw), (*Bacillus subtilis* + straw), (*Mariannaea camptospora* + straw). The highest decrease in ADL value was observed in treatment (consortia + straw) 7.48%, 6.26%, 5.33%, 3.99% at 15, 30, 45, and 60 DAI, respectively (Table 5). Effective lignin degradation was observed, supporting findings of Islam et al. (2021), who reported *Bacillus subtilis* aids lignin degradation (9.47%). The lignin content in the cell walls of rice straw undergoes enzymatic degradation, reorganization, expansion, and breakdown by lignocellulolytic microbes, facilitating biomass decomposition (Liu H. et al., 2021; Liu Y. et al., 2021; Sruthy et al., 2023).

##### 3.5.3.3 Cellulose reduction

Cellulose content represents the difference between the ADF and ADL content. After 60 DAI cellulose reduction showed similar patterns with highest decrease observed in the (consortia + straw) declining from 50.84 to 29.55%. Furthermore, followed by (*Mariannaea camptospora* + straw) 30.52%, (*Bacillus subtilis* + straw) 31.29% and (*Pseudomonas aeruginosa* + straw) 33.09% in contrast with (control) 48.46% (Table 6). Microbial consortia enhance enzymatic activity and, facilitate rapid cellulose degradation (Kimura et al., 2005). Recent studies by Datsomor et al. (2022) demonstrated that microbial-treated straw exhibits higher degradation rates, reinforcing the potential of lignocellulolytic microbes in agricultural waste management.

**Table 6:** Effect of single and consortia of lignocellulolytic microbial inoculation on cellulose content and decomposition rate of straw under *in vitro* conditions.

Treatments	Cellulose (%)					Decomposition rate (%)	
	0 DAI	15 DAI	30 DAI	45 DAI	60 DAI	0 DAI	60 DAI
T_1_	50.84	50.3029^a^ ± 0.0599	49.8033^a^ ± 0.1236	49.1782^a^ ± 0.1452	48.455^a^ ± 0.0766	0	33.32^e^ ± 0.2462
T_2_	50.84	48.7308^bc^ ± 0.7212	42.9169^c^ ± 0.4625	37.6338^bc^ ± 0.7651	31.291^c^ ± 0.1317	0	54.4^c^ ± 0.1633
T_3_	50.84	49.2631^b^ ± 0.7017	44.3392^b^ ± 0.4625	38.2999^b^ ± 0.3926	33.0873^b^ ± 0.385	0	50.1025^d^ ± 0.0818
T_4_	50.84	48.295^c^ ± 0.7341	42.4633^c^ ± 0.3945	37.4233^c^ ± 0.3089	30.515^d^ ± 0.2187	0	58.4^b^ ± 0.3266
T_5_	50.84	47.2173^d^ ± 0.4462	41.382^d^ ± 0.5274	34.1736^d^ ± 0.3605	29.5479^e^ ± 0.4181	0	66.6525^a^ ± 0.1226

Values mentioned are the mean of four replications; DAI, days after inoculation. Different superscript letters (a–e) indicate statistically significant differences (*P* = 0.05) among Control (Straw without microbe), Bacillus subtilis + Straw, Pseudomonas aeruginosa + Straw, Mariannaea camptospora + Straw, Microbial consortia + Straw. Values are means ± standard deviation.

#### 3.5.4 Integrated structural analysis

The significant decrease in the ADF, ADL, and cellulose content of the straw during decomposition is due to the hydrolysis of the substrate's cellulose and lignin into simpler sugars. Treatment of straw with the microbial (consortia + straw) resulted in the most significant reduction in ADF, ADL, and cellulose content. This enhanced degradation is attributed to synergistic interactions among microorganisms within the consortium, which perform more efficiently than individual strains in terms of growth, metabolic functions, and enzymatic activity (Kimura et al., 2005). The decreased in values was higher under *in vitro* conditions because the optimum temperature, moisture and environmental conditions were favorable for microbial enzyme activity and resulted in more degradation.

### 3.6 Environmental implications and biotechnological applications

#### 3.6.1 Straw decomposition analysis and soil health enhancement

The maximum decomposition rate (*k*-value) was recorded with the microbial (consortia + straw) 66.65%, and (*Mariannaea camptospora* + straw) 58.40%, demonstrating superior straw degradation relative to individual microbial treatments. Control therapy 33.32%, presented the lowest rate. This indicates that microbial consortia considerably improve straw decomposition through synergistic interactions among various microorganisms that generate complementary enzymes for organic matter degradation (Sen et al., 2010; Goyal and Sindhu, 2011) (Table 6). The pot experiment revealed that straw treated with the microbial consortia achieved highest degradation efficiency and superior growth characteristics of rice crops. This suggests that straw decomposition enhances soil fertility and nutrient availability, thereby promoting plant growth (Liu H. et al., 2021; Liu Y. et al., 2021). These results underscore the efficacy of microbial consortia for the decomposition of rice straw. Straw decomposition and higher soil vitality resulting from microbial treatment facilitate sustainable agriculture operations by alleviating straw management difficulties and increasing crop yield. Furthermore, isolates such as *Bacillus subtilis* and *Pseudomonas aeruginosa*, known for their plant growth promoting rhizobacteria (PGPR) capabilities and biocontrol potential, contribute to the observed superior growth characteristics of rice crops, positioning this microbial solution within the frameworks of regenerative agriculture. Microbial inoculant treatments, especially (consortia + straw), showed notable enzyme activity. Elevated enzyme activities (cellulase, amylase, and protease) in the soil treated with consortia signify improved organic matter breakdown and nutrient cycling (Dash et al., 2022). Similarly, Sharma et al. (2024) demonstrated positive correlations between microbial consortia and soil enzyme activities during crop residue decomposition. These residual enzymatic activities suggest continued benefits for soil organic matter cycling, nutrient availability, and microbial community structure beyond immediate straw decomposition.

#### 3.6.2 Climate change mitigation potential

The accelerated aerobic decomposition process significantly reduces methane emissions associated with anaerobic straw decomposition in waterlogged fields. The 66.65% decomposition efficiency suggests potential for substantial reduction in agricultural residue burning, which contributes approximately 23% of particulate matter pollution in rice-growing regions and releases significant quantities of CO_2_, CH_4_, and N_2_O. Our study revealed that selected microbial isolates possessed significant lignocellulolytic capabilities, primarily through the production of cellulase, xylanase, and ligninase enzymes. Recent studies highlight the importance of lytic polysaccharide monooxygenases (LPMOs) in augmenting these enzymes. LPMOs oxidatively cleave crystalline cellulose and synergize with glycoside hydrolases, significantly enhancing biomass hydrolysis efficiency (Singh et al., 2024).

#### 3.6.3 Circular economy integration and value addition

##### 3.6.3.1 Biofertilizer production potential

Solid-state fermentation (SSF) has been increasingly recognized for its superior enzyme production potential using agro-industrial residues. Our SSF results demonstrated high enzyme titers, validating earlier findings that SSF is an ideal method for enzyme generation in resource-limited settings (Mattedi et al., 2023). The enhanced nutrient profile (N: 1.62%, P: 0.28%, K: 1.83%) of consortium-treated straw creates opportunities for commercial biofertilizer production.

##### 3.6.3.2 Bio-based product development

In addition to the agronomic and environmental benefits, valorization of rice straw through microbial decomposition also opens avenues for bio-based product development. As Mandal et al. (2024) highlighted agri-waste such as rice straw serves as a valuable substrate for the microbial synthesis of bio plastics, notably polyhydroxyalkanoates (PHAs), using strains like *Pseudomonas aeruginosa*, which was also part of our microbial consortium. These insights not only reinforce the ecological potential of microbial lignocellulose decomposition but also suggest the feasibility of coupling straw degradation with bioplastic production as part of an integrated waste valorization strategy aligned with circular bio economy principles.

The implications of microbial decomposition extend beyond mere straw clearance. Valorization of decomposed biomass into bioethanol, organic acids, or microbial fertilizers offers circular economy solutions and aligns with global sustainability targets. Strategies like biofilm-based degradation and enzyme enhancement via genetic engineering or nanotech further amplify decomposition efficiency. Furthermore, microbial decomposition of lignocellulosic biomass like rice straw not only aids in nutrient recycling but also opens up pathways for its conversion into value-added products (Kumar et al., 2023; Ganguly et al., 2025b). Strategies like microbial valorization of persistent substrates, biofilm-based degradation, and enzyme enhancement via genetic engineering or nanotech further amplify decomposition efficiency (Ganguly et al., 2025a; Janeeshma et al., 2024). Recent studies have shown that bacterial strains such as *Pseudomonas aeruginosa*, identified in our study, can be harnessed for the biosynthesis of biodegradable plastics like polyhydroxyalkanoates (PHAs) from agri-waste substrates. This provides a compelling case for integrating microbial decomposition with bioplastic production to achieve sustainable and circular waste management (Mandal et al., 2024; Mitra et al., 2024).

## 4 Conclusion

This study emphasizes the effective creation and utilization of a lignocellulolytic microbial consortium for the sustainable decomposition of rice straw. The consortium, consisting of microbial isolates exhibiting elevated ligninase and cellulase activities, showed enhanced effectiveness in degrading complex lignocellulosic substrates relative to individual treatments or untreated controls. Under *in vitro* conditions investigations have validated an accelerated decomposition rate, augmented soil enzyme activity, and raised microbial biomass carbon, which collectively promoted soil fertility and rice crop growth. This method provides an environmentally viable alternative to straw burning, reducing pollution and greenhouse gas emissions while fostering nutrient recycling and sustainable agriculture. Future investigations should concentrate on transitioning these optimized laboratory findings to *in-situ* field trials to validate efficacy under variable environmental parameters (e.g., moisture, temperature fluctuations, and complex microbial competition). Furthermore, enhancing the consortia composition and environmental parameters should be prioritized to optimize field performance. These findings will enable the integration of microbial consortia into extensive agricultural residue management systems to address critical environmental sustainability issues.

## Data Availability

The original contributions presented in the study are included in the article/supplementary material, further inquiries can be directed to the corresponding author.
